# The effect of silver nanoparticles (AgNPs) on proliferation and apoptosis of *in ovo* cultured glioblastoma multiforme (GBM) cells

**DOI:** 10.1186/s11671-015-0823-5

**Published:** 2015-03-01

**Authors:** Kaja Urbańska, Beata Pająk, Arkadiusz Orzechowski, Justyna Sokołowska, Marta Grodzik, Ewa Sawosz, Maciej Szmidt, Paweł Sysa

**Affiliations:** Division of Histology and Embryology, Department of Morphological Sciences, Faculty of Veterinary Medicine, Warsaw University of Life Sciences-SGGW, Nowoursynowska 159, 02-776 Warsaw, Poland; Department of Physiological Sciences, Faculty of Veterinary Medicine, Warsaw University of Life Sciences-SGGW, Nowoursynowska 159, 02-776 Warsaw, Poland; Electron Microscopy Platform, Mossakowski Medical Research Center, Polish Academy of Sciences, Pawińskiego 5, 02-106 Warsaw, Poland; Division of Nanobiotechnology, Faculty of Animal Science, Warsaw University of Life Sciences-SGGW, Ciszewskiego 8, 02-786 Warsaw, Poland

**Keywords:** Glioblastoma multiforme, Silver nanoparticles, *In ovo* model, Proliferation, Apoptosis

## Abstract

Recently, it has been shown that silver nanoparticles (AgNPs) provide a unique approach to the treatment of tumors, especially those of neuroepithelial origin. Thus, the aim of this study was to evaluate the impact of AgNPs on proliferation and activation of the intrinsic apoptotic pathway of glioblastoma multiforme (GBM) cells cultured in an *in ovo* model. Human GBM cells, line U-87, were placed on chicken embryo chorioallantoic membrane. After 8 days, the tumors were divided into three groups: control (non-treated), treated with colloidal AgNPs (40 μg/ml), and placebo (tumors supplemented with vehicle only). At the end of the experiment, all tumors were isolated. Assessment of cell proliferation and cell apoptosis was estimated by histological, immunohistochemical, and Western blot analyses. The results show that AgNPs can influence GBM growth. AgNPs inhibit proliferation of GBM cells and seem to have proapoptotic properties. Although there were statistically significant differences between control and AgNP groups in the AI and the levels of active caspase 9 and active caspase 3, the level of these proteins in GBM cells treated with AgNPs seems to be on the border between the spontaneous apoptosis and the induced. Our results indicate that the antiproliferative properties of silver nanoparticles overwhelm proapoptotic ones. Further research focused on the cytotoxic effect of AgNPs on tumor and normal cells should be conducted.

**Electronic supplementary material:**

The online version of this article (doi:10.1186/s11671-015-0823-5) contains supplementary material, which is available to authorized users.

## Background

Numerous complications are associated with the use of conventional anticancer drugs, including insolubility in water, rapid clearance, and lack of selectivity, resulting in non-specific toxicity regarding normal cells and insufficient dose of drug delivered to the cancer cells [1]. Nanoparticles (NPs) exploit biological pathways to achieve payload delivery to cellular and intracellular targets, including transport across the blood-brain barrier (BBB). The ability of these carriers to overcome BBB appears to be enabled by receptor-mediated endocytosis through brain endothelial cells. The possibility of employing nanoparticles for delivery of proteins and other macromolecules across the BBB suggests that this technology holds great promise for non-invasive therapy of the CNS diseases [2], including neoplasms. Because NPs have the affinity to acidic environment, which characterized tumor tissue, it is believed that selective targeting strategies with NPs facilitate more effective cancer detection and treatment with minimized side effects to normal cells [1]. Non-cytotoxic doses of silver nanoparticles (AgNPs) have been recently extensively investigated due to their capacity to induce the expression of genes associated with impaired cell cycle progression, DNA damage, and apoptosis in human cells [3]. According to AshaRani and colleagues [3], AgNPs induced DNA damage leading to cell cycle arrest in G2/M phase and enhanced apoptosis of tumor cells, including glioblastoma multiforme (GBM).

Most studies focused on the efficacy of anticancer drugs in the treatment of GBM are conducted *in vitro*. Despite the fact that *in vitro* studies are characterized by simple methodology, this experimental model has important limiting factors, related mainly to the bioavailability and cell biodistribution of anticancer drugs. Moreover, tumor cells growing in such conditions lack the architectural and cellular complexity of *in vivo* tumors, so it is impossible to recreate the interaction between the tumor and its host [4].

The influence of antitumor drugs on glioblastoma, as well as other tumor, cell activity can be also investigated in *in vivo* models: animal model (mice, rats) and *in ovo* model. The advantages of an *in ovo* model compared to animal models included simple methodology of tumor cell implantation, economical reasons, and high survival rate of embryos. Moreover, being naturally immunodeficient, the chick embryo may accept transplantation from various tissues and species, without immune responses. In contrast to standard mouse models, most tumor cells implanted on the chicken chorioallantoic membrane (CAM) blood vessels survive without cell damage. An *in ovo* model allows a rapid development and vascularization of forming tumors. Tumors growing in an *in ovo* model are visible within 2 to 5 days after tumor cell implantation, compared to *in vivo* models, where tumors form within 3 to 6 weeks. A possibility to conduct daily non-invasive observations of growing tumors is also a great advantage of this experimental model [5]. Thus, an *in ovo* model can be successfully applied in oncological studies [6]. It has been used to culture several types of tumors and to study their growth rate, angiogenic potential, and metastatic capability [5].

Given the promising data concerning the AgNPs utilization in anticancer therapy of CNS tumors and the reliable utility of an *in ovo* model in oncological studies, the aim of this study was to evaluate the impact of AgNPs on proliferation and apoptosis activity of GBM cells cultured in an *in ovo* model, including the levels of active caspase 9 and active caspase 3. According to the authors’ knowledge, no such studies have been previously conducted.

## Methods

### Cell culture

Human glioblastoma multiforme cells, line U-87 MG (ATCC, No HTB-14), were incubated in a Sanyo CO_2_ incubator (SANYO Electric Biomedical Co., Ltd, Osaka, Japan) under standard conditions (37°C, 5% CO_2_, 95% humidity), in Dulbecco’s modified Eagle’s medium (Sigma Aldrich Chemical Co., St Louis, MO, USA) with the addition of inactive fetal bovine serum (10% *v*/*v*) (Sigma Aldrich) and antibiotics such as 50 U/ml of penicillin and 50 μg/ml of streptomycin (Sigma Aldrich). Before transplantation to chicken embryos, the cell culture was treated with trypsin/EDTA (0.25% *v*/*v*, Sigma Aldrich). Then, cells were centrifuged (1,200 rpm/5 min/400 *g*) and dispersed in culture medium in a concentration of 5 × 10^6^ cells/20 μl per egg. To determine the viability of GBM cells at the day of cell implantation on CAM, trypan blue assay was used.

### Chicken embryo culture

The experiments were performed on 75 fertilized chicken eggs of Ross 308 line. The eggs were obtained from a breeding farm ‘Drobiarstwo-działy specjalne, Lidia i Henryk Malec’ (Dębówka, Poland). The eggs were incubated in an incubator ALMD-1 N3-7 (F.H.U. Waleński, Gostyn, Poland) with automatic egg rotation (one full rotation per hour) at 37°C and 70% humidity.

### Implantation of GBM cells on CAM of chicken eggs

The process of implantation of GBM cells was performed on the sixth day of egg incubation in a laminar hood cabinet under sterile conditions. A 0.5-cm^2^ hole was cut in the egg’s shell after its cleaning with potassium permanganate (Hasco Lek, Wroclaw, Poland). The internal parchment membrane of the air chamber was dissected, and a small silicone ring was placed on the blood vessel area, and 5 × 10^6^ of tumor cells suspended in 20 μl drop of medium were placed into the silicone ring. Then, the holes in the eggshell were protected by air-permeable adhesive tape (Polopor, 3 M Vicoplast S.A., Wroclaw, Poland), and eggs were moved back to the incubator (37°C and 70% humidity) without rotation.

### Silver nanoparticles

**Figure 1:**
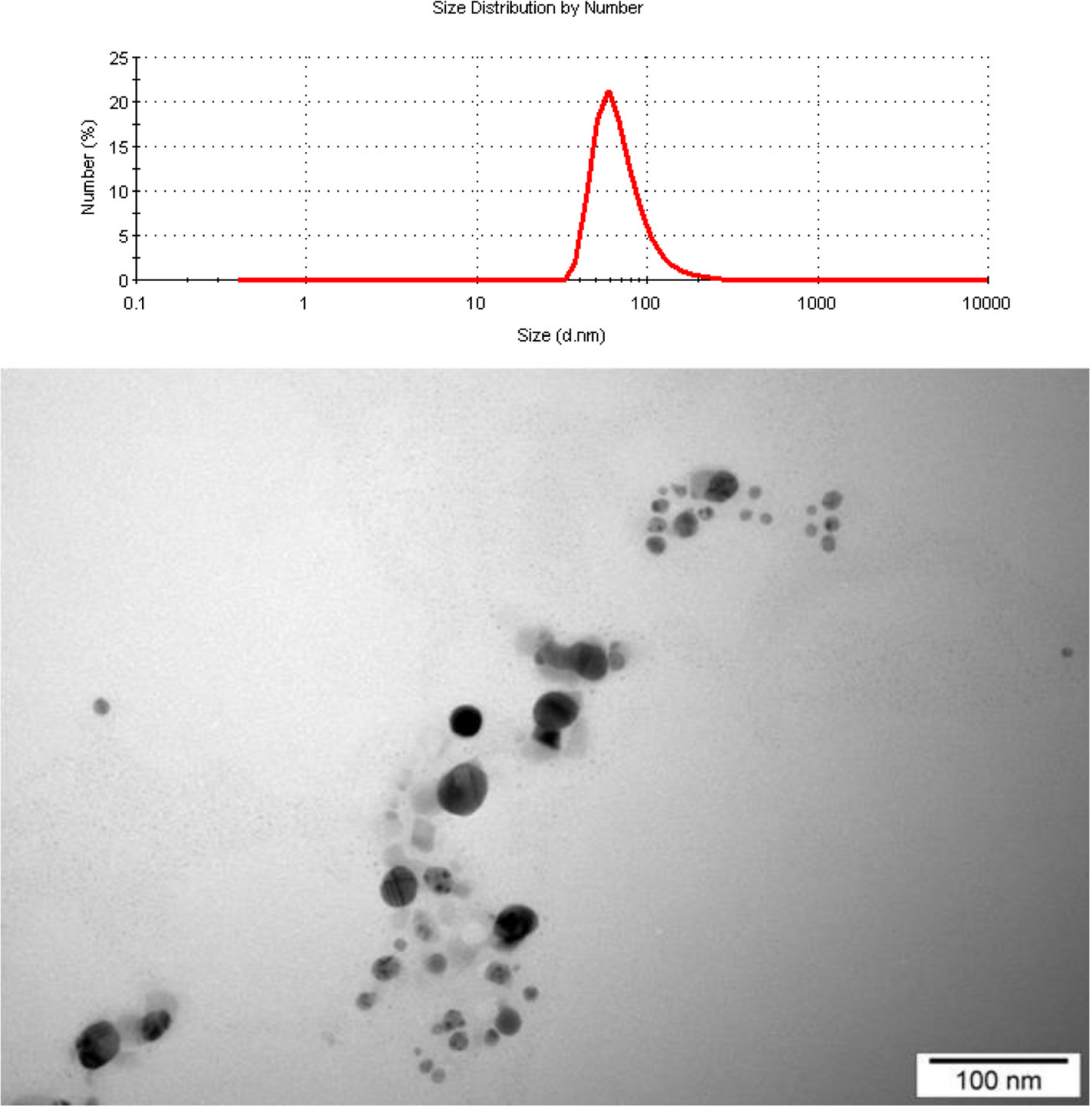
**Size distribution and TEM image of silver nanoparticles.**

The hydrocolloid of nano-Ag (AgNPs) obtained from Nano-Tech (Warsaw, Poland) was produced by an electric non-explosive patented method (patent number US2009020364 A1) from high-purity metals (99.9999%) and high-purity demineralized water [7]. The physical and chemical properties of AgNPs were characterized by Chwalibog et al. [8]. The shape and size of NPs were inspected with a Jeol JEM-1220 transmission electron microscope (TEM) at 80 KeV (JEOL, Tokyo, Japan), with a Morada 11 megapixel camera (Olympus Soft Imaging Solutions GmbH, Münster, Germany) (Figure 1). Samples of Ag for TEM were prepared by placing droplets of hydrocolloids onto formvar-coated copper grids (Agar Scientific Ltd, Stansted, UK). Nanoparticles of Ag were mostly spherical and polydispersed. The stability of the colloidal dispersions of the nanoparticles (zeta potential) was measured by the electrophoretic light-scattering method with a Zetasizer Nano ZS, model ZEN3500 (Malvern Instruments, Worcestershire, UK). The zeta potential of Ag nanoparticles was −36.4 mV, and the average diameter of particles was 70 nm (Figure 1). AgNPs were dissolved in ultra-pure water (Milli-Q water system, Millipore Corp., Billerica, MA, USA).

### Application of AgNPs and placebo solutions

On the 14th day of the eggs’ incubation, the tumors were randomly divided into three groups: control group (C; non-treated tumors), AgNPs group (AgNPs; tumors treated with colloidal AgNPs), and placebo group (Pl; tumors supplemented with ultra-pure water). The AgNPs solution used in the experiment (40 μg/ml) was prepared by diluting the AgNano stock solution (50 μg/ml) in ultra-pure water. Concentration of AgNPs solution was based on literature data and *in vitro* cytotoxic assays (unpublished data). After dilution, AgNP solution was incubated in bath sonication (3 cycles × 15 min) and prepared freshly before use.

AgNPs solution (50 μl) was injected into each tumor, and an additional 100 μl of solution was applied to the tumor surface and the blood vessels adjacent to the tumor. Similarly, in the placebo group, ultra-pure water was used. All procedures were performed under sterile conditions with a non-pyrogenic disposable insulin syringe (Polfa Lublin, S.A., Lublin, Poland).

### Tumor isolation

After the 18th day of incubation, the chicken embryos were sacrificed and the tumors were isolated. The number of isolated GBM was as follows: control group *n* = 20, AgNPs group *n* = 20, and placebo group *n* = 15. Each tumor was fixed in 4% buffered formalin. In nine cases (three tumors from each group), one part was fixed in formalin and second was frozen at −80°C. According to Balke and colleagues’ protocol (2010), only tumors with a minimum 2-mm diameter and a visible area of vascularization were taken for further analysis [9]. In the case of a few tumors grown in one egg, the tumor with the highest diameter located to the nearest silicone ring was chosen for analysis.

### Histological and immunohistochemical analyses

After fixation in formalin, tumors were embedded in paraffin, cut into 4-μm slides, and stained by the hematoxylin-eosin method (H&E) and immunohistochemically with anti-Ki-67, anti-active caspase 9, and anti-active caspase 3 antibodies. All immunohistochemical procedures were performed according to the manufacturer’s protocols. Endogenous peroxidase activity was blocked by 5-min incubation in H_2_O_2_ solution. Antigen unmasking was performed by microwave (two cycles: 7 and 5 min, 700 W) or water bath (20 min, 98°C). Sections were cooled for 20 min, rinsed with tris-buffered saline plus Tween 20 (TBST) (Dako, Golstrup, Denmark), and then incubated with primary antibody for 1 h at room temperature or overnight at 4°C. Dako REAL™ EnVision™ Detection System, Peroxidase/DAB^+^, Rabbit/Mouse visualization system was used for antigen detection. Details of the primary antibodies and antigen retrieval methods used in immunohistochemical evaluation are presented in Table 1.

**Table 1:** **Primary antibodies and antigen retrieval methods used in immunohistochemical evaluation**

**Immunohistochemistry**				
**Primary antibody**	**Clonality**	**Antigen unmasking**	**Dilution**	**Producer**
Ki-67, clone MIB-1	Monoclonal	Microwave, citrate buffer, pH 6.0	1:50^a^	Dako
Active caspase 3	Polyclonal	Water bath, citrate buffer, pH 6.0	1:800^b^	Cell Signaling
Active caspase 9	Polyclonal	Microwave, EDTA buffer, pH 8.0	1:40^a^	Novocastra

^a^1-h incubation, RT.

^b^Overnight incubation, 4°C.

### Assessment of cell proliferation

Tumor cell proliferation was estimated in the sections stained with H&E and immunohistochemically with anti-Ki-67 antibody. The proliferative activity was estimated on the basis of the mitotic index (MI) and proliferative index (PI) in each specimen. MI was assessed as the mean number of metaphase and anaphase nuclei in ten visual fields, in triple counting (H&E, at × 400 magnification).

PI was defined as the number of Ki-67-positive GBM cells in 10^3^ tumors cells (at × 1,000 magnification). Marginal and necrotic areas of the tumors were excluded from analyses.

### Assessment of cell apoptosis

Tumor cell apoptosis was estimated by the terminal deoxynucleotidyl transferase dUTP nick-end labeling (TUNEL) method and immunohistochemically with anti-active caspase 9 and anti-active caspase 3 antibodies. The apoptotic activity of GBM cells was defined as the apoptotic index (AI). Apoptotic cells were detected by the TUNEL method with the ApopTag® Peroxidase In Situ Apoptosis Detection Kit (Merck Millipore Headquarters, Billerica, MA, USA) according to the manufacturer’s protocol. AI was calculated as a percentage of apoptotic cells or apoptotic bodies in 10^3^ tumor cell population, without marginal areas of the tumors (at × 1,000 magnification).

Active caspase 9 (casp9I) and active caspase 3 (casp3I) indices were defined as the percentage of positive cells in 10^3^ tumor cells (at × 1,000 magnification). According to Kobayashi and colleagues [10], only tumors with casp9I or casp3I >10.00% were considered as active caspase-positive. Marginal and necrotic areas of the tumors were excluded from analyses.

### Western blot analysis

Total protein extraction from tumors was performed with the tissue homogenizer X360 (Ingenierburo CAT M. Zipperer, Staufe, Germany) at 4°C with RIPA buffer (1×PBS, 10 ml/l Igepal CA-630, 5 g/l sodium deoxycholate, 1 g/l SDS; Sigma Aldrich) supplemented with 0.4 mM of PMSF, 10 μg/ml of aprotinin, and 10 μg/ml of sodium orthovanadate (Sigma Aldrich). After centrifugation (10,000 rpm, 5 min, 300 *g*, 4°C), extractions were divided into equal volumes and stored at −80°C until analysis. For protein quantification in the whole-cell lysates, the protein-dye-binding method of Bradford [11] with commercially available reagents was used (Bio-Rad Laboratories, Hercules, CA, USA). An equal protein amount (25 μg/well) was subjected to 12% SDS-PAGE electrophoresis at 150 V. Then, proteins were transferred at 100 V for 2 h to polyvinylidene difluoride (PVDF) membranes. Further, non-specific binding was blocked via membrane incubation with 5% non-fat dry milk in TBST for 1 h at RT. Membranes were immunoblotted overnight at 4°C with antibodies against active forms of caspase 9 or caspase 3. A list of all primary and secondary antibodies used in the Western blot method is provided in Table 2. Next, membranes were incubated with the secondary antibody conjugated with HRP (Table 2). Membranes were also probed with goat polyclonal anti-actin antibody to normalize protein levels. The blots were developed by the enhanced chemiluminescence (ECL) detection system (Amersham International, Aylesbury, UK) according to the manufacturer’s protocol. After exposure, photographs were taken with a Kodak DC 290 zoom digital camera and scanned and analyzed by the Kodak EDAS 290/Kodak 1D 3.5 system. Three independent experiments were performed for each protein.

**Table 2:** **Primary and secondary antibodies used in Western blot method**

**Western blot**					
**Primary antibody**	**Dilution**	**Producer**	**Secondary antibody**	**Dilution**	**Producer**
Active caspase 3^a^	1:1,000	Cell Signaling	Anti-rabbit	1:10,000	Santa Cruz
Active caspase 9^a^	1:1,000	Cell Signaling	Anti-rabbit	1:10,000	Santa Cruz
Actin^a^	1:200	Santa Cruz	Anti-goat	1:5,000	Santa Cruz

^a^Polyclonal antibody.

### Statistical analysis

Statistical analyses of immunohistochemical data were performed with Statistica 8.0 PL software, StatSoft Inc. The results were expressed as mean ± SD, minimum, maximum, and median values. Continuous variables were compared by a nonparametric Mann-Whitney *U* test. Statistical significance was interpreted as highly significant at *P* ≤ 0.001.

Results obtained from Western blot analysis were statistically evaluated with one-way ANOVA and Tukey’s comparison multiple range tests with GraphPad Prism™ version 4.0 software (GraphPad Software Inc., San Diego, USA). The results were expressed as mean ± SE, and a value of *P* ≤ 0.05 was considered to be significant.

## Results

### The influence of AgNPs on GBM cells proliferation

**Figure 2:**
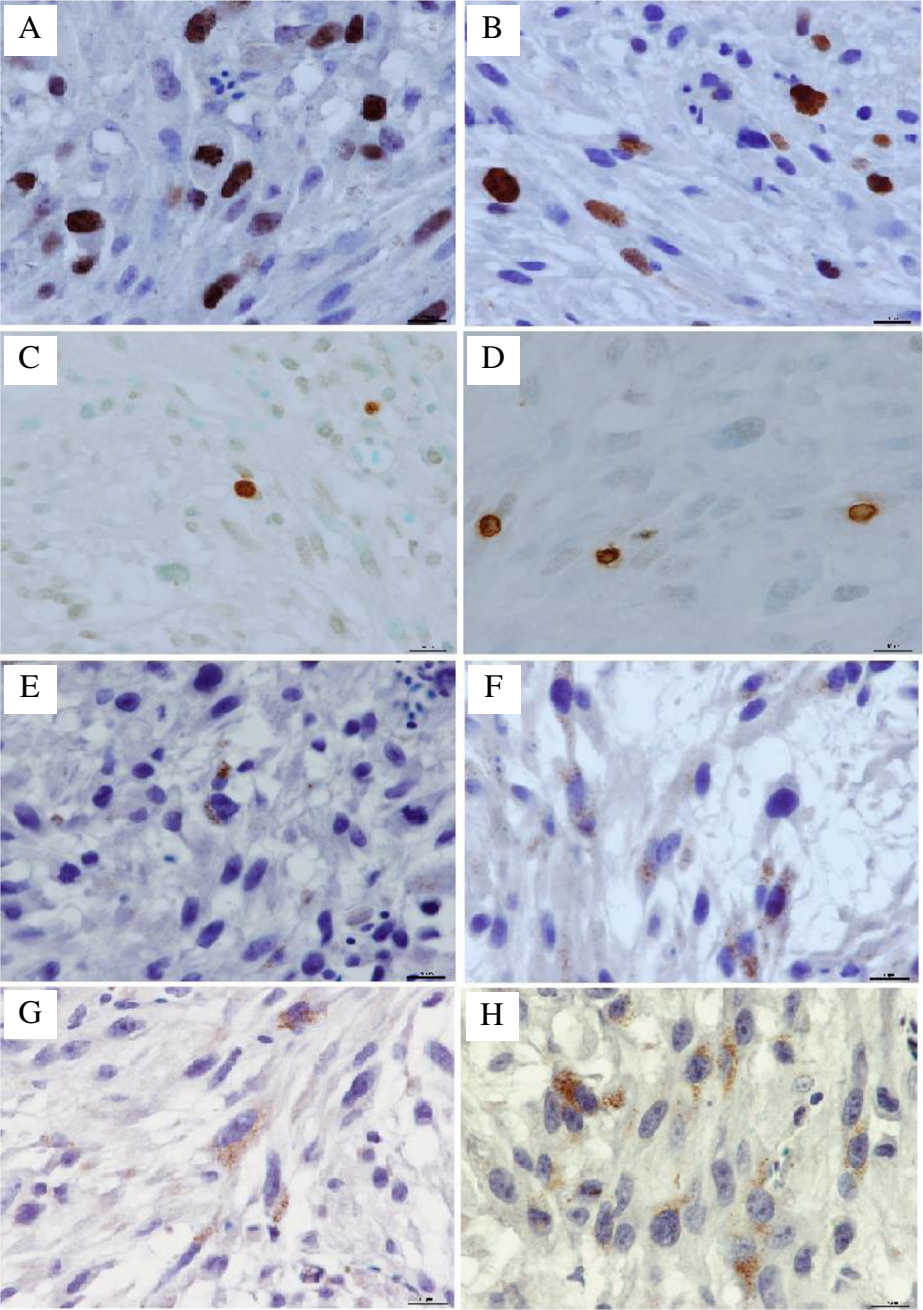
**The influence of AgNPs on GBM cells proliferation and apoptosis indices.** Ki-67 expression in GBM cells from control group **(A)** and from GBM cells treated with AgNPs **(B)**. Apoptotic cells in GBM from control group **(C)** and from GBM treated with AgNPs **(D)**. Active caspase 9 expression in GBM cells from control group **(E)** and from GBM cells treated with AgNPs **(F)**. Active caspase 3 expression in GBM cells from control group **(G)** and from GBM cells treated with AgNPs **(H)**, bar = 10 μm.

**Figure 3:**
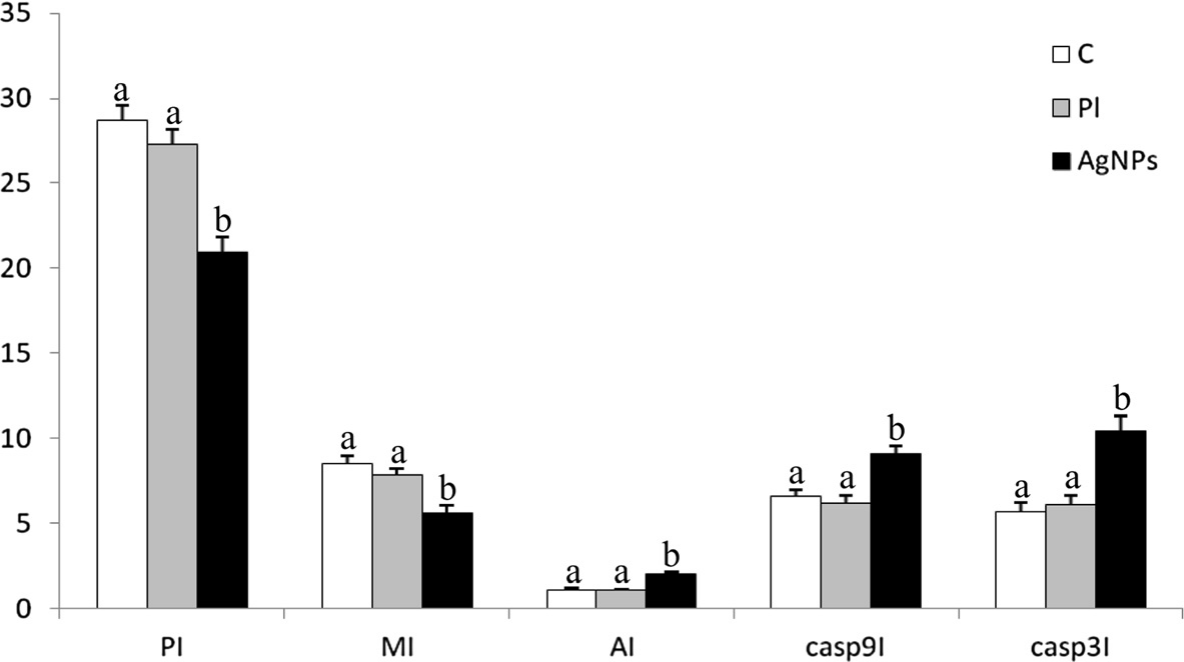
**Comparing mean values of all immunohistochemically/histologically examined parameters in tumors from control, placebo, and AgNPs groups.** Letters (a, b) mean highly statistically significant differences, *P* ≤ 0.001.

Examination of GBM cells revealed that the PI in control untreated tumors ranged from 21.00 to 36.00% with a mean of 28.72% ± 0.85% (Figure 2A), which was in agreement with the results obtained for the placebo group. In the placebo group, Ki-67^+^ cells ranged from 21.40 to 35.70% (with a mean of 27.29% ± 0.89%). In the AgNPs-treated group, the PI index was significantly lower (*P* ≤ 0.001) compared to the control and placebo groups and ranged from 13.70 to 31.00% (mean 20.93% ± 0.69%) (Figure 2B). In the control group, MI ranged from 4.10 to 12.20 (mean 8.54 ± 0.48). In the placebo group, the percentage of GBM cells in metaphase and anaphase was 5.25 to 11.23 (mean 7.89 ± 0.36), comparable to the results for the tumors from the control group. The MI of tumors from the AgNPs group ranged from 1.90 to 10.83 (mean 5.62 ± 0.43). All changes caused by AgNPs administration led to a significant reduction of GBM cells in the M phase compared with cells from control and placebo groups (*P* ≤ 0.001). Results of MI and PI mean values of all groups are summarized in Figure 3.

### The influence of AgNPs on GBM cells apoptosis

The administration of AgNPs did not induce cell apoptosis; however, the AI values, as well as casp9I and casp3I, were significantly higher in the AgNPs-treated tumors. AI in the control group ranged from 0.8 to 2.30% (mean 1.12% ± 0.09%) (Figure 2C) and was fit into the values of the spontaneous apoptosis intensity rate. In the placebo group, the number of positive stained cells was similar and ranged from 0.60 to 1.30% (mean 1.07% ± 0.05%). In the AgNPs group, the AI values were also within the spontaneous apoptosis values and ranged from 1.40 to 3.30% (Figure 2D). However, the mean AI value (2.02% ± 0.12%) obtained for this group was statistically higher (*P* ≤ 0.001) than in control and placebo groups.

Activation of apoptosis was confirmed by the detection of active caspase 9 and active caspase 3 level *via* immunohistochemistry and Western blot. Immunohistochemical analysis showed cytoplasmic expression of active caspase 9 with moderate staining intensity. Casp9I was low, independently from the experimental group. Immunohistochemical study showed that all cases from the control group were considered as active caspase 9 negative (Figure 2E). The percentage of active caspase 9^+^ cells ranged from 4.90 to 9.80% (mean 6.64% ± 0.30%). Similar results were obtained in the placebo group, with the exception of one tumor with more than 10% of caspase 9-positive cells (casp9I was from 2.80 to 10.80% with the mean 6.14 ± 0.49). After AgNPs treatment, the percentage of caspase 9^+^ cells significantly increased (*P* ≤ 0.001) and ranged from 4.90 to 12.30% (Figure 2F). However, mean casp9I was only 9.13 ± 0.48.

**Figure 4:**
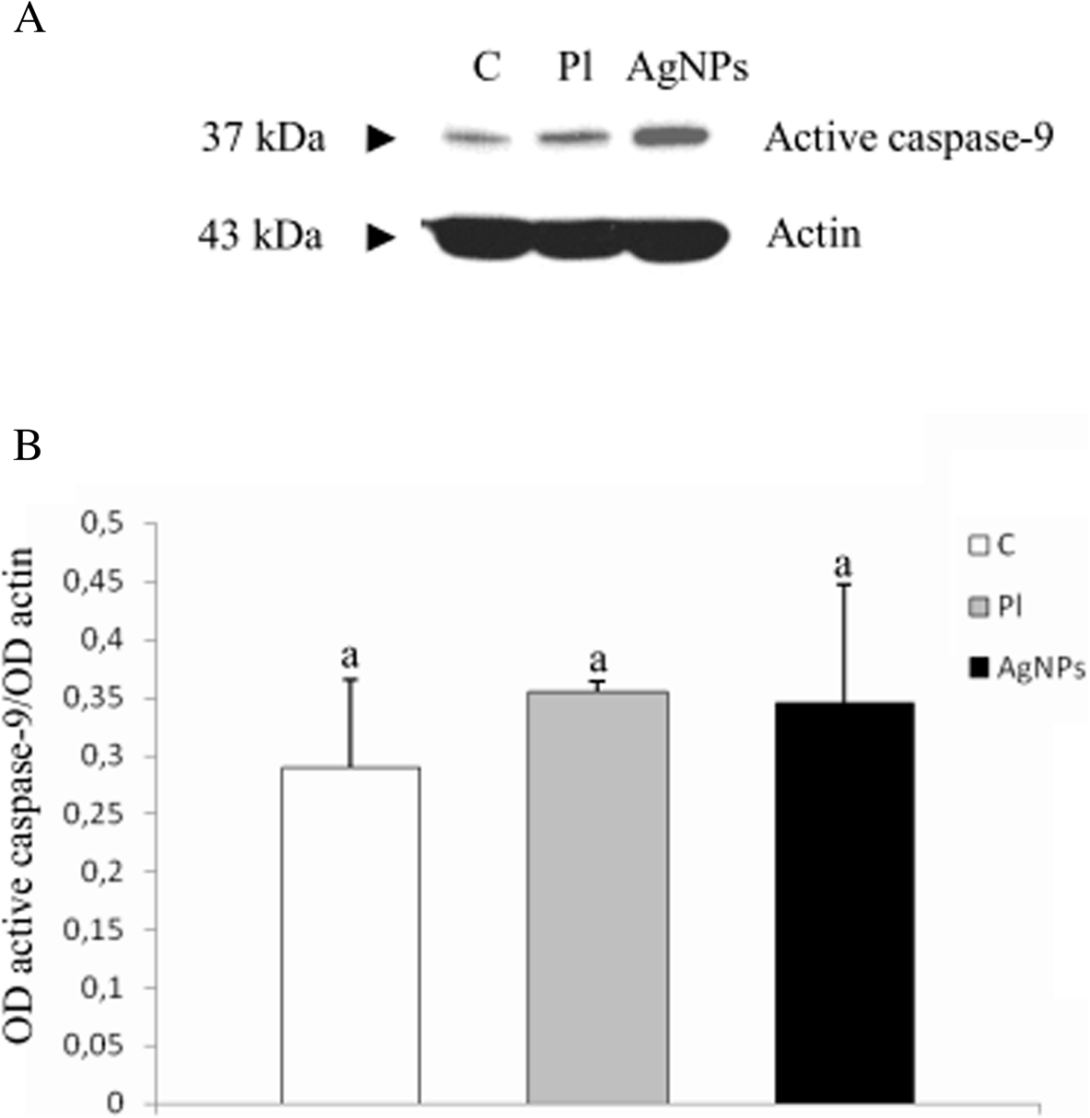
**The level of active caspase 9 in GBM cells cultured in an** ***in ovo*** **model. (A)** Western blot analysis of the level of active caspase 9 in GBM cells from control group (C), placebo group (Pl), and AgNPs group (AgNPs). **(B)** Graph showing the optical density values (optical density, OD) obtained for the different bands, expressed in relative terms, defined as the ratio of OD values obtained for band representing active caspase 9 and OD values obtained for actin band. Letters (a, a) mean no statistically significant differences, *P* ≤ 0.05. The obtained results are the mean of three independent experiments.

Western blot analysis confirmed that the level of active caspase 9 expression was higher in AgNPs-treated and placebo groups than in the control group; however, the differences were not statistically significant (*P* ≤ 0.05, Figure 4).

**Figure 5:**
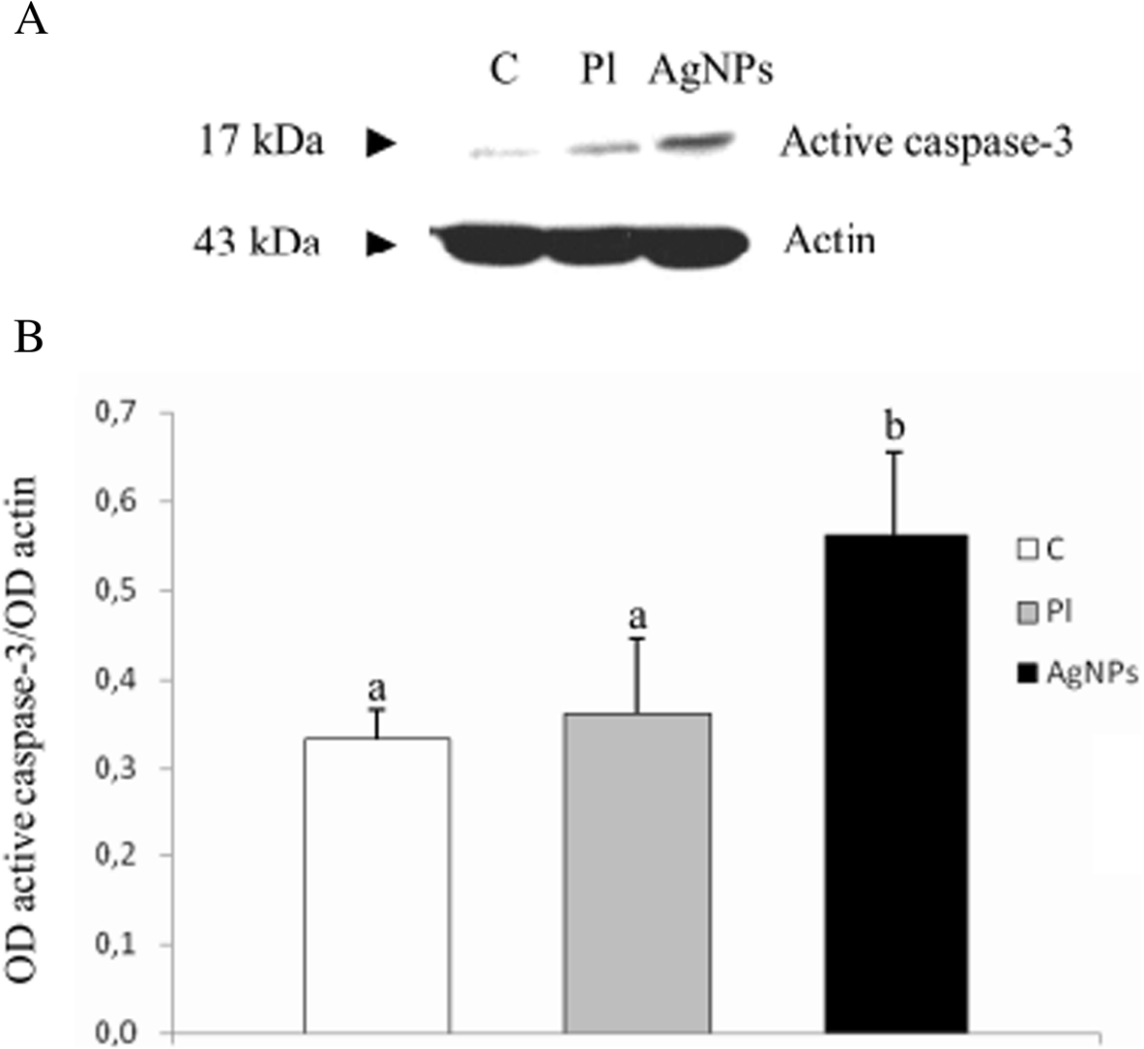
**The level of active caspase 3 in GBM cells cultured in an** ***in ovo*** **model. (A)** Western blot analysis of the level of active caspase 3 in GBM cells from control group (C), placebo group (Pl), and AgNPs group (AgNPs). **(B)** Graph showing the optical density values (optical density, OD) obtained for the different bands, expressed in relative terms, defined as the ratio of OD values obtained for band representing active caspase 3 and OD values obtained for actin band. Letters (a, b) mean highly statistically significant differences, *P* ≤ 0.05. The obtained results are the mean of three independent experiments.

The intensity of anti-active caspase 3 immunolabeling was stronger than with anti-caspase 9 antibody. Results obtained from immunochemical analysis of the presence of active caspase 3 were in agreement with those described for active caspase 9. In the control group, the percentage of active caspase 3^+^ cells (Figure 2G) was similar to the placebo group (Figure 2H) (2.80 to 11.30%, mean 5.65% ± 0.55%, and 3.40 to 11.30%, mean 6.07 ± 0.61, respectively). In the AgNPs group, casp3I was 5.50 to 19.50% (Figure 2H). The statistical analysis showed that the mean number of casp3I in the AgNPs group (10.46% ± 0.88%) was higher than in others (*P* ≤ 0.001), as shown in Figure 4. However, the lower limits of casp3I in AgNPs-treated tumors were similar to those in the control and placebo groups. When analyzed by Western blot, the level of active caspase 3 was shown to increase in AgNPs-treated tumors compared with other groups and the difference was statistically significant (*P* ≤ 0.05, Figure 5).

Results of AI, casp9I, and casp3I mean values of all groups are summarized in Figure 3. The detailed immunohistochemical and histological results for control, placebo, and AgNPs groups are given in Additional file 1: Tables S1, S2, and S3 (see in Additional file 1), respectively.

## Discussion

Nanoparticles are attractive material for medical purposes because of their unique features, including large surface to mass ratio. A relatively large (functional) surface is able to bind, adsorb, and carry other compounds such as drugs, probes, and proteins, e.g., ligands, which recognize receptors of the target cells [12]. Due to the slightly acidic pH of tumor cells, the release of silver ions from AgNPs is higher in tumor cells compared to normal cells. Thus, NPs can help enhance the accumulation of drug within the tumor while limiting accumulation in healthy organs. AgNPs loaded with different chemotherapeutic drugs induce selective toxicity and enhance efficacy of anticancer drugs, which enable to reduce their dosage [13]. Additionally, NPs modulate cellular and humoral immune responses. Recently, some studies in rodents have examined the NPs distribution, pharmacokinetics, and drug delivery into the brain and found that NPs with a diameter greater than 100 to 150 nm tend to accumulate in tumors because of their higher extravasation in comparison with normal vasculature [14-16].

The cytostatic effect of silver on cancer cells is the result of the active physico-chemical interaction of silver atoms with the functional groups of intracellular proteins as well as nitrogen bases and phosphate groups of DNA [17]. In our study, AgNPs administration caused a significant decrease of proliferation in GBM cells. Both examined proliferation indices (PI and MI) were significantly lower in the AgNPs group in comparison with control and placebo groups. The PI values of tumors treated by AgNPs were similar to glioblastoma multiforme with long-term survival (6.00 to 20.00%) [18], and the most often diagnosed spontaneous GBM, which causes death within less than 1 year, has higher PI values: 25.00% to 30.00% [19,20]. Values obtained for spontaneous glioblastoma correspond to the PI we obtained for control and placebo groups. The lower values of examined proliferation markers may be a result of AgNPs accumulation in the nucleus of GBM cells, where it causes chromosome instability and mitotic arrest. Hence, tumor cells cease to proliferate [3]. Another explanation of AgNP-mediated inhibition of cell division could be AgNPs’ interaction with structure and functions of actin cytoskeleton, involved in cell division signaling cascades [3]. Results obtained in our study confirm the antiproliferative properties of AgNPs in GBM cells and show that AgNPs could possess potential benefits in terms of the anticancer treatment of GBM patients (related to prolongation of overall survival) as similar observations were published previously [3,21,22]. Lara-González and colleagues [22] discovered that the administration of AgNPs to tumor-bearing mice at the time of tumor injection caused significantly increased survival, compared with controls. This effect was more potent than after vincristine treatment.

The intensity of apoptosis also has a major impact on tumor growth. Studies have revealed a high frequency of apoptosis in spontaneously regressing tumors and tumors treated with cytotoxic agents. Thus, the estimation of apoptosis in tumor cells (AI), together with PI, is an important marker of tumor development and has prognostic significance [23]. There is a wide variation in the extent of spontaneous apoptosis not only between different tumor types but also within in a given tumor type. The spontaneous AI, in GBM and other tumors, measured by the TUNEL method, does not exceed 10.00% in any of them [24].

In our study, the mean AI of GBM cells cultured *in ovo*, in control, and placebo groups was 1.12% and 1.07%, respectively. Results obtained for any experimental group were within the limits for spontaneous GBM presented by other authors, who estimated the number of apoptotic cells in GBM patients as less than 1.50% and even 0.00% in some GBM cases [25]. Apoptosis is also particularly important for the development of effectiveness of anticancer drugs as they restore the ability of cancer cells to self-eliminate [23] or enhance thermo-sensitivity of neoplasm cells [26]. A large number of stimuli, including various chemotherapeutic agents, ultraviolet and γ-irradiation, heat, osmotic imbalance, high calcium concentration, and nitrogen oxide can induce apoptosis [24]. There is some information about the induction of apoptosis by AgNPs [26,27]. Proapoptotic properties of AgNPs were also verified in our study. Further studies are, however, needed.

Our results showed that AgNPs administration caused a statistically significant increase of GBM AI values. This effect could result from the generation of reactive oxygen species (ROS) by AgNPs. The active surface of AgNPs can directly induce the generation of original free radicals, and the dissolution of AgNPs into Ag ions. It caused damage to DNA and disruption of mitochondrial membrane potential, released cytochrome c, and led to mitochondrial-dependent apoptosis [3,28,29]. Although AgNPs administration caused a statistically significant increase in apoptosis, all obtained results of tumors from the AgNPs group are in agreement with the results for apoptosis in spontaneous GBM, which was at the level of <3.00% [30-33]. It is well known that malignant tumor cells, such as GBM, are extremely resistant to apoptosis and particularly resistant to radiation and chemotherapy. It can be explained by low levels of spontaneous apoptosis, which might correlate with enhanced resistance to cytotoxic treatment strategies [34]. This may explain the slight increase of AI values in GBM treated with AgNPs compared with the control group (2.02% *vs.* 1.12%).

Results of the other study have shown that the proapoptotic effect of silver nanoparticles can be achieved by its repetitious administration [22]. Our observations suggest that during single AgNPs administration, the higher AgNPs concentration could potentially enhance their proapoptotic effect. Finally, AgNPs could be conjugated with chemotherapeutic agents used in GBM treatment, which allow a lower dose of the anticancer drugs and minimize the cytotoxic influence on normal cells.

There is only one publication about the immunohistochemical characterization of caspase level in spontaneous GBM. Bodey and colleagues [35] estimated the percentage of casp 9^+^ in tumor cells at the level of 10%, which is consistent with the results presented in our study (mean value of casp9I was 6.64%, 6.14%, and 9.13% in control, placebo, and AgNPs groups, respectively). Bodey and colleagues [35] assessed the staining intensity as very strong to strong. In our study, immunohistochemical analysis showed that the expression of caspase 9 in control and placebo tumors was comparable and the staining intensity was from strong to weak, and only in one case in the placebo group was casp9I considered as positive. Several mechanisms blocking the activation of apoptotic pathways, including caspase 9 activation blocking, have been postulated in GBM cells, which could explain the low level of this protein in GBM [36,37]. These hypotheses seem to be confirmed in our studies, where low casp9I values were obtained for all analyzed tumors. Low expression of caspase 9 was confirmed also by Zarnescu and colleagues [38], who estimated the expression of caspase 9 in U-87 glioblastoma xenografts. Caspase 9 overexpression and its activation lead to apoptosis. Since the majority of anticancer strategies initiate apoptosis through caspase 9 activation, the modulation of caspase 9 expression may be exploited by designing new ways to control apoptosis in malignant tumors, including GBM [39]. There are a lot of publications about the interactions between different types of nanoparticles and tumor cells which have shown that titanium dioxide nanoparticles as well as curcumin-loaded nanoparticles cause intrinsic-mediated apoptosis in human cells and that caspase 9 plays a critical role in this process [40,41]. However, none of these publications focused on the activation of caspase 9 in GBM. In our study, the level of active caspase 9 protein in control GBM was very low. AgNPs caused an increase of active caspase 9 expression in GBM cells. The majority of cases defined as positive were found in just this group. It seems that NPs could significantly contribute to the development of new approaches of drug delivery in cancer and provide a platform for combined therapeutics with subsequent monitoring of response [42]. However, this hypothesis requires more extensive studies.

Apart from caspase 9, which is an initiator caspase, the most important role in the apoptosis pathway activation is played by caspase 3, an executioner caspase. Caspase 3 is expressed in normal and neoplastically transformed human cells; however, the expression of this caspase in CNS neurons is low or is not detected at all [35]. The level of the active form of caspase 3 has been examined in many types of cancer in correlation with its histological grade of malignancy and may be used as a prognostic marker of the patient’s overall survival [43]. In our study, caspase 3 has been shown in the cytoplasmic staining pattern; however, a tendency to translocation from the cytoplasm to the cell nuclei may occur [35]. Schiffer and colleagues [33] showed positive staining of active caspase 3 in nuclei, cytoplasms, or in both cell compartments [33]. The percentage of GBM cells with positive nuclear reactions can reach 10% of all tumor cells [35]. Some regulators of DNA metabolism can be activated by caspase 3, which subsequently translocates to the nucleus [33]. Apart from the fact that the presence of active caspase-3 in the nucleus starts the execution phase of apoptosis, such localization may affect the evasion of the immune system by the tumor cells [35].

There are large divergences in casp3I values in spontaneous GBM. In some studies, the percentage of caspase 3^+^ cells ranges from 7.36 to 59.52% (mean 17.67%) [44], whereas in other cases it ranges from 0.70 to 71.60% (mean 32.60%) [45]. In our study, the immunohistochemical staining and densitometric analysis of electropherograms of Western blot have shown that the expression of the active form of caspase 3 in control and placebo groups were lower than in the AgNPs group. However, the mean value of casp3I in tumors treated with AgNPs was still much lower compared with the spontaneous glioblastomas, 50.00% [35], even in the AgNPs group. However, the methodology used by the cited authors differs from that presented in our work. Results obtained by Bodey and colleagues [35] were determined by estimation. Tirapelli and colleagues [44] counted the casp 3^+^ cells in two visual fields per slide with the highest number of positively staining cells. Kobayashi and colleagues [10] counted positive cells in a population of 500 cells, and in our study, 1,000 cells from each specimen were counted. Results obtained by other authors indicate that in GBM cells, the fraction of casp 3^+^ cells is too low to activate the apoptotic program [45].

In our study, the value of casp3I significantly increased after AgNPs treatment of GBM cells, which was confirmed immunohistochemically and by the Western blot method. It may indicate, according to Piao and colleagues [29], that AgNPs have the power to induce cell death through the activation of caspase-dependent pathways. The same results obtained by Gurunathan and colleagues [46] showed that during treatment of MDA-MB-231 cells with AgNPs, the level of caspase 3 increased to a level comparable with that of caspase-3 activation [46]. It was also confirmed by Satapathy and colleagues [47]. One of the reasons for the death of cancer cells with AgNPs is the release of silver ions from NPs. The generation of silver ions from AgNPs is the main culprit in terms of the formation of oxidative stress that activates caspase 3 [48].

## Conclusions

The results of our study indicate that AgNPs can influence biological activity of U-87 cells (defined as their proliferative and apoptotic indices). AgNPs inhibit proliferation of GBM cells and have proapoptotic properties. However, the level of active caspase 3 and active caspase 9 in GBM cells after AgNPs treatment seems to be on the border between spontaneous and induced apoptosis. For these reasons, research focused on the effects of AgNPs on the proliferation and apoptotic activity of GBM should be continued.

## Additional file

Additional file 1: Table S1. Results of immunohistochemical staining for all examined parameters in particular cases of GBM from control group. **Table S2.** Results of immunohistochemical staining for all examined parameters in particular cases of GBM from placebo group. **Table S3.** Results of immunohistochemical staining for all examined parameters in particular cases of GBM from AgNPs-treated group.

## Abbreviations

AgNPs: Silver nanoparticles
AI: Apoptotic index
BBB: Blood-brain barrier
CASP3I: Active caspase 3 index
CASP9I: Active caspase 9 index
CNS: Central nervous system
GBM: Glioblastoma multiforme
H&E: Hematoxylin-eosin method
MI: Mitotic index
NPs: Nanoparticles
PI: Proliferative index
TUNEL: Terminal deoxynucleotidyl transferase dUTP nick end labeling
