# Supplementary material for: The effect of silver nanoparticles (AgNPs) on proliferation and apoptosis of in ovo cultured glioblastoma multiforme (GBM) cells
Source: Nanoscale Res Lett. 2015 Mar 1;10:98. doi: 10.1186/s11671-015-0823-5 (PMC4385140; doi:10.1186/s11671-015-0823-5)
Supplement: Additional file 1: Table S1. — Results of immunohistochemical staining for all examined parameters in particular cases of GBM from control group. Table S2. Results of immunohistochemical staining for all examined parameters in particular cases of GBM from placebo group. Table S3. Results of immunohistochemical staining for all examined parameters in particular cases of GBM from AgNPs-treated group. [file 11671_2015_823_MOESM1_ESM.doc]

**Additional file**

Table S1. Results of immunohistochemical staining for all examined parameters in particular cases of GBM from control group

The values of all examined parameters for control group (C): PI - proliferative index, MI - mitotic index, AI - apoptotic index, casp9I - active caspase 9 index, casp3I - active caspase 3 index.

| **Control group (C)** | | | | | | |
| --- | --- | --- | --- | --- | --- | --- |
| **No.** | **Case number** | **PI (%)** | **MI** | **AI (%)** | **casp9I (%)** | **casp3I (%)** |
| 1 | 1819 | 28.40 | 8.80 | 0.90 | 5.80 | 6.70 |
| 2 | 1841 | 27.20 | 12.20 | 1.20 | 7.10 | 7.00 |
| 3 | 1822 | 36.00 | 10.06 | 0.90 | 7.80 | 4.10 |
| 4 | 1839 | 28.50 | 8.40 | 1.00 | 5.30 | 5.30 |
| 5 | 1824 | 26.50 | 10.80 | 1.20 | 5.65 | 4.50 |
| 6 | 1838 | 24.00 | 7.87 | 0.90 | 7.15 | 3.50 |
| 7 | 1825 | 25.00 | 10.3 | 0.80 | 7.30 | 4.80 |
| 8 | 1852 | 26.50 | 9.63 | 2.30 | 5.80 | 6.20 |
| 9 | 1851 | 36.00 | 10.05 | 1.00 | 9.30 | 3.90 |
| 10 | 1849 | 26.80 | 7.15 | 1.00 | 4.90 | 11.30 |
| 11 | 1846 | 33.90 | 11.60 | 1.10 | 6.50 | 8.30 |
| 12 | 1844 | 26.70 | 7.86 | 1.10 | 7.35 | 3.60 |
| 13 | 1843 | 28.30 | 8.40 | 1.00 | 5.20 | 3.20 |
| 14 | 9GK5 | 28.30 | 9.10 | 1.10 | 4.90 | 6.50 |
| 15 | 1847 | 29.70 | 7.00 | 1.20 | 6.85 | 11.00 |
| 16 | 1911 | 32.20 | 6.20 | 1.90 | 9.80 | 3.80 |
| 17 | 1910 | 32.00 | 9.00 | 0.90 | 7.50 | 5.20 |
| 18 | 1894 | 31.40 | 4.10 | 1.10 | 5.40 | 3.40 |
| 19 | 1896 | 25.20 | 8.10 | 0.80 | 6.40 | 2.80 |
| 20 | 1909 | 21.80 | 4.20 | 1.00 | 6.90 | 8.00 |

Table S2. Results of immunohistochemical staining for all examinated parameters in particular cases of GBM from placebo group

| **Placebo group (Pl)** | | | | | | |
| --- | --- | --- | --- | --- | --- | --- |
| **No.** | **Case number** | **PI (%)** | **MI** | **AI (%)** | **casp9I (%)** | **casp3I (%)** |
| 1 | 1820 | 27.30 | 11.23 | 1.00 | 5.15 | 10.10 |
| 2 | 1840 | 22.70 | 8.67 | 1.20 | 5.30 | 5.60 |
| 3 | 1835 | 26.40 | 9.17 | 1.00 | 6.35 | 5.80 |
| 4 | 1831 | 31.30 | 6.63 | 1.50 | 10.80 | 5.00 |
| 5 | 9GPl3 | 27.30 | 7.46 | 0.80 | 5.70 | 3.40 |
| 6 | 1890 | 26.10 | 7.76 | 1.20 | 6.40 | 6.90 |
| 7 | 1884 | 28.90 | 8.70 | 1.00 | 8.40 | 4.15 |
| 8 | 1887 | 26.90 | 7.40 | 1.10 | 2.80 | 4.50 |
| 9 | 1886 | 29.00 | 7.00 | 0.60 | 4.20 | 4.80 |
| 10 | 1908 | 35.70 | 7.26 | 0.90 | 6.40 | 5.00 |
| 11 | 1885 | 21.40 | 8.66 | 1.10 | 3.90 | 4.30 |
| 12 | 1897 | 26.90 | 6.53 | 1.10 | 7.10 | 4.10 |
| 13 | 1888 | 28.20 | 8.60 | 1.20 | 6.00 | 11.30 |
| 14 | 1907 | 23.40 | 5.26 | 1.30 | 6.40 | 8.30 |
| 15 | 1883 | 27.90 | 7.95 | 1.10 | 7.30 | 7.90 |

The values of all examined parameters for placebo group (Pl): PI - proliferative index, MI - mitotic index, AI - apoptotic index, casp9I - active caspase 9 index, casp3I - active caspase 3 index.

Table S3. Results of immunohistochemical staining for all examined parameters in particular cases of GBM from AgNP treatment group

| **AgNP group (AgNP)** | | | | | | |
| --- | --- | --- | --- | --- | --- | --- |
| **No.** | **Case number** | **PI (%)** | **MI** | **AI (%)** | **casp9I (%)** | **casp3I (%)** |
| 1 | 1830 | 21.40 | 4.23 | 3.30 | 12.00 | 14.80 |
| 2 | 1845 | 20.15 | 4.90 | 1.90 | 10.70 | 9.60 |
| 3 | 1829 | 20.60 | 4.27 | 1.90 | 11.30 | 8.70 |
| 4 | 1850 | 20.40 | 8.10 | 1.50 | 8.60 | 5.50 |
| 5 | 1828 | 22.20 | 6.13 | 1.40 | 10.00 | 10.00 |
| 6 | 1827 | 31.00 | 6.00 | 1.60 | 10,60 | 19.50 |
| 7 | 1900 | 24.30 | 5.20 | 2.80 | 6,70 | 5.70 |
| 8 | 1903 | 17.00 | 1.90 | 2.40 | 4,90 | 5.50 |
| 9 | 1902 | 20.90 | 5.06 | 2.10 | 5,30 | 6.90 |
| 10 | 1906 | 21.60 | 5.93 | 2.80 | 12,30 | 13.40 |
| 11 | 1878 | 19.80 | 5.66 | 1.40 | 7.90 | 16.20 |
| 12 | 1898 | 20.10 | 6.00 | 2.50 | 8.30 | 14.20 |
| 13 | 1882 | 20.20 | 10.83 | 2.20 | 8.80 | 12.40 |
| 14 | 1904 | 27.70 | 4.02 | 1.60 | 7.10 | 7.90 |
| 15 | 1880 | 13.70 | 5.06 | 2.20 | 9.20 | 9.90 |
| 16 | 1879 | 18.70 | 5.46 | 1.60 | 11.80 | 12.00 |
| 17 | 1905 | 19.30 | 3.30 | 2.30 | 8.20 | 7.20 |
| 18 | 1901 | 25.60 | 8.43 | 1.50 | 9.80 | 5.80 |
| 19 | 1881 | 18.30 | 6.07 | 1.70 | 11.00 | 11.00 |
| 20 | 1877 | 15.70 | 5.80 | 1.70 | 8.10 | 12.90 |

The values of all examined parameters for silver nanoparticle treated group (AgNP): PI - proliferative index, MI - mitotic index, AI - apoptotic index, casp9I - active caspase 9 index, casp3I - active caspase 3 index.
